# Sedation Strategies for Awake Carotid Endarterectomy: An Exploratory Retrospective Study Comparing Dexmedetomidine and Remifentanil

**DOI:** 10.3390/clinpract16020023

**Published:** 2026-01-23

**Authors:** Rosanna Carmela De Rosa, Antonio Romanelli

**Affiliations:** 1 Department of Anaesthesia and Intensive Care, Cotugno Hospital, AORN dei Colli, Via Quagliariello, 80131 Naples, Italy; 2 Department of Anaesthesia and Intensive Care, AOU San Giovanni di Dio e Ruggi D’Aragona, Via San Leonardo, 84131 Salerno, Italy; antonioromanelli86@gmail.com

**Keywords:** awake surgery, carotid endarterectomy, dexmedetomidine, remifentanil, local anesthesia, and sedation strategies

## Abstract

**Background:** Awake carotid endarterectomy (CEA) under local anesthesia demands an optimal sedation strategy that ensures patient comfort while preserving the ability for real-time neurological assessment. Dexmedetomidine (DEX) and remifentanil (REMI) are widely used agents, but direct comparisons in this setting remain scarce. **Methods:** Exploratory, retrospective, single-center study of awake CEA (March–July 2019). DEX or REMI infusions were titrated to a Richmond Agitation–Sedation Scale (RASS) of −1 to −2. Outcomes were sedation failure (RASS ≥ +2 despite maximum infusion rate), bradycardia, hypotension, and neurologic events. Statistical analyses used χ^2^ test (categorical variables) and Student’s *t*-test or Mann–Whitney test (continuous variables). Associations were assessed with Firth’s logistic regression (univariable and bivariate models), reporting odds ratios (OR) with 95% confidence intervals (CI_95%_). Trends in the Bispectral Index (BIS), hemodynamic, and respiratory parameters were assessed using two-way repeated-measures Analysis of Variance (ANOVA). A *p*-value < 0.05 was considered significant. **Results:** Fifty-two patients were included (DEX = 25; REMI = 27). DEX group showed more frequent sedation failure (32.0% vs. 3.7%; *p* = 0.020), bradycardia (36.0% vs. 3.7%; *p* = 0.009), and hypotension (28.0% vs. 0%; *p* = 0.011). DEX was associated with increased risk in sedation failure (OR 8.58, CI_95%_ 1.70–85.81), bradycardia (OR 10.17, CI_95%_ 2.05–101.21), and hypotension (OR 22.30, CI_95%_ 2.46–2959.60); the direction of associations remained consistent in bivariate models adjusted for baseline confounders. ANOVA showed group-by-time interactions for BIS, heart rate, mean arterial pressure, and end-tidal CO_2_. No intraoperative complications or adverse outcomes were observed. **Conclusions:** In this retrospective cohort of awake CEA, DEX was associated with higher rates of sedation failure and hemodynamic adverse events compared with REMI, without an apparent impact on procedural success. Given non-random allocation and baseline imbalances, these findings are hypothesis-generating and warrant confirmation in larger, robust, and prospective studies.

## 1. Introduction

Carotid endarterectomy (CEA), the most frequently performed vascular surgical procedure, is a safe and effective technique to reduce the risk of stroke in patients with high-grade internal carotid artery (ICA) stenosis. During CEA, the main goal is to prevent cerebral ischemia resulting from hypoperfusion after carotid clamping or embolic events during clamping or unclamping. Monitoring awake patients under local anesthesia (LA) is the most reliable method for detecting intraoperative neurological deficits [1,2,3]. The significant advantage of the awake patient is the ability to continuously monitor neurologic function by simply talking to the patient and asking to perform basic tasks with the contralateral hand, rather than using EEG or other continuous neuromonitoring techniques [4,5].

However, during awake CEA, head position, near-face drapes, immobility, and pain can induce anxiety and discomfort in the patient. The anesthesiologist should provide an adequate sedation level that accurately balances the patient’s tolerability to the procedure and the prompt execution of a neurological examination. It is essential to avoid too-deep sedation that can alter the immediate execution of a neurological exam and determine depression of respiratory drive with apnea and desaturation. Moreover, anesthesiologists should prevent excessive changes in hemodynamic parameters that can alter cerebral perfusion.

Remifentanil (REMI), a short-acting synthetic opioid, plays a crucial role in awake CEA by allowing patients to remain conscious and enabling direct observation of neurological function. The rapid onset and offset are suitable for managing intraoperative stress and providing optimal analgesia without compromising recovery time [6]. Although REMI has been shown to provide stable hemodynamic conditions during surgery [7], it may also induce respiratory depression. Therefore, careful monitoring and management of ventilation are required, particularly in elderly patients with comorbidities [8,9].

Another suitable choice is dexmedetomidine (DEX). DEX, a highly selective α_2_-adrenergic agonist, causes sedation without suppressing the respiratory drive, as opposed to opioids. In addition, the patient sedated with DEX can be easily awakened, allowing the execution of simple orders, in contrast to propofol and midazolam. However, its administration is associated with significant hemodynamic alterations, including hypotension and bradycardia, which could alter cerebral perfusion [10,11,12].

To the best of our knowledge, the literature lacks a comprehensive comparison of the effects of REMI and DEX in this surgical setting. This exploratory study compares the effectiveness and safety of REMI and DEX as sedative agents during awake CEA with cervical plexus block. Furthermore, we explored the differences in parameter trends noted during specific phases of the surgical procedure.

## 2. Materials and Methods

This was an exploratory, retrospective study conducted at a single center (Monaldi Hospital, AORN dei Colli, Naples, Italy). We retrospectively identified all consecutive patients who underwent awake CEA under LA between March and July 2019 by reviewing institutional anesthesia and surgical records. Written informed consent for the surgical procedure and anesthesia was obtained as part of routine clinical care, in accordance with applicable law. All data were collected and analyzed in anonymized form, and patient privacy was rigorously protected in accordance with current national legislation, including the General Data Protection Regulation of the European Union (No. 2016/679) and the Italian Legislative Decrees (No. 196/2003 and No. 101/2018). The study was conducted in accordance with the International Conference on Harmonisation Good Clinical Practice guidelines and the provisions of the 2008 Declaration of Helsinki. The present study was reported in accordance with the Guidelines for Strengthening the Reporting of Observational Studies in Epidemiology (STROBE).

The inclusion criterion was primary CEA under LA. The indications were symptomatic ICA stenosis ≥ 70% regardless of age or asymptomatic ICA stenosis > 70% in patients younger than 75 years. The only exclusion criterion was the need to perform CEA with general anesthesia. During the study period, the same attending anesthesiologist provided anesthetic management for all procedures, and all patients were operated on or supervised by a single experienced vascular surgeon.

Standard intraoperative monitoring included five electrodes electrocardiography, pulse oximetry (SpO_2_), and non-invasive blood pressure. In addition, the level of sedation was monitored using the Bispectral Index (BIS™, Medtronic, Minneapolis, MN, USA). All patients received O_2_ (4.0 L/min) by nasal cannula with end-tidal CO_2_ (EtCO_2_) monitoring. We monitored cerebral oxygenation using near-infrared spectroscopy (NIRS, INVOS-5100C, Somanetics Corporation, Medtronic, Minneapolis, MN, USA). NIRS is a simple, non-invasive, real-time technique that estimates regional cerebral oxygen saturation (rSO_2_) by measuring the relative absorption of near-infrared light by oxygenated and deoxygenated hemoglobin. Adhesive sensors were applied to the frontal region after skin preparation, according to the manufacturer’s instructions, to provide continuous monitoring of frontal rSO_2_, reflecting changes in cerebral oxygenation in territories predominantly supplied by the anterior and middle cerebral arteries. The system uses a light emitter and two detectors at different distances to reduce contamination from superficial tissues and to preferentially sample deeper signals, thereby improving the estimation of cerebral rSO_2_.

All patients were not premedicated. Once the patient was correctly positioned on the operating table, basal heart rate (HR), mean arterial pressure (MAP), SpO_2_, BIS, and end-tidal CO_2_ (EtCO_2_) values were noted. The LA technique involved ultrasound-guided cervical infiltration using a 0.5% ropivacaine solution, up to a total dose of 150 mg. Successful LA was defined as the absence of pain at the skin incision and throughout the procedure.

Due to the retrospective nature of the study, no allocation strategy was implemented. In routine practice during the study period, the two sedation regimens (DEX and REMI) were used in an alternating fashion across operating sessions, so that consecutive sessions generally adopted different regimens. No formal randomization sequence, allocation concealment, or stratification was applied. Before LA, the patient started a continuous infusion of REMI (starting dose 0.05 mcg/kg/min) or DEX (starting dose 0.8 mcg/kg/h, without the loading dose) on a peripheral vein placed at the entry in the operating room. The dose was adjusted, increasing or decreasing by 20% from the baseline infusion rate, until we reached our “sedation goal”, defined as a Richmond Agitation–Sedation Scale (RASS) value ranging from −1 to −2. If, despite the maximum dose of REMI (0.15 mcg/kg/min) or DEX (1.4 mcg/kg/hour), the RASS value was ≥+2, the anesthesiologist reported this event as “failed sedation” and administered a sedative drug (midazolam, 1 mg). The RASS ≥ +2 threshold reflects a clinically meaningful level of agitation in awake CEA, where loss of cooperation and excessive movement can affect intraoperative neurological monitoring and operative safety, justifying the use of rescue sedation.

All patients underwent standard CEA by patch closure of the arteriotomy. The screening for shunt placement was based on awake testing and rSO_2_ drop performed immediately after clamping and during the first three minutes of clamping. The anesthesiologist asked the patient to squeeze a noise-making object in the contralateral hand to the clamped carotid and to perform simple commands (e.g., open and close the eyes, move the upper and lower extremities, show the tongue and teeth). An abnormal awake test, defined as the onset of a new neurological symptom or an altered state of consciousness, was used as a criterion for shunt placement (Pruitt–Inahara carotid shunt with T-Port LeMaitre Vascular Inc., Burlington, MA, USA). Basal rSO_2_ was calculated as the value three minutes before clamping when heparin was administered. In all patients, the percentage of rSO_2_ drop after cross-clamping was calculated using the following equation:

↓rSO_2_% = (rSO_2α_ − rSO_2β_/rSO_2α_) × 100

with rSO_2α_ as the basal regional oxygen saturation and rSO_2β_ being the regional oxygen saturation after carotid cross-clamping. In no-shunted patients, rSO_2β_ was the value after three minutes of clamping, while in shunted patients, it was the value just before shunt insertion. A drop of rSO_2_ > 20% was considered a cut-off value of hypoperfusion [13,14]. To enhance cerebral perfusion, the intraoperative blood pressure was maintained at preoperative levels, and hypotension was avoided, especially during the clamping phase, by administering a vasoconstrictor to titrate arterial pressure to the baseline level.

In case of the development of hemodynamic changes due to sedative infusion, the anesthesiologist followed this protocol:

- When bradycardia (HR < 50 bpm) occurred, the anesthesiologist administered atropine (0.5 mg) and/or isoprenaline (10.0 mcg bolus) iv;
- When hypotension (MAP < 65 mmHg) occurred, the anesthesiologist administered noradrenaline (10.0 mcg iv).

In the case of bradycardia and/or hypotension, the sedative infusion was reduced by 20%. Every hemodynamic effect was noted.

For every patient, we collected the following variables:

- Sex, age, weight, comorbidities, and ASA status;
- Clamping time, the occurrence of positive awake test, NIRS drop, and the need for shunt placement;
- The occurrence of sedation failure, bradycardia, and hypotension.

Sedative dose, MAP, HR, SpO_2_, EtCO_2_, and BIS values were noted at specific time points:

- T_0_: starting sedative infusion;
- T_1_: at the skin incision;
- T_2_: at carotid clamping;
- T_3_: at carotid declamping;
- T_4_: at the end of surgery.

Categorical variables were reported as absolute values and percentages (%). Continuous variables were reported as mean ± standard deviation (SD) or as the median and first and third quartiles (q_1_–q_3_), depending on distribution (tested with the Shapiro–Wilk test). Minimum and maximum were also reported.

We divided our population into two groups: REMI Group and DEX Group. The difference in categorical variables was tested with the χ^2^ test. Differences in continuous variables were tested with the Student *t*-test (or the Welch test for heteroscedasticity) or the Mann–Whitney test (U-test). The association between sedative regimens and study outcomes (sedation failure, intraoperative bradycardia, hypotension, positive awake test, and NIRS drop) was evaluated using Firth’s penalized logistic regression. We performed both univariable models and adjusted (bivariable) models, in which the sedative agent was entered together with potential confounders identified from baseline imbalances. Results are reported as odds ratios (OR) with 95% confidence intervals (CI_95%_) and corresponding *p*-values.

To analyze trends in BIS, HR, MAP, SpO_2_, and EtCO_2_ across time points and between groups, and to explore the interaction between time and groups, we performed a two-way repeated-measures Analysis of Variance (ANOVA). Two-way ANOVA was performed on both absolute values and baseline-standardized changes. Standardized changes were expressed as relative change from baseline, computed as (T_x_ − T_0_)/T_0_, and reported as a percentage. Effect size was measured with Generalized Eta-squared (η^2^), and defined as small (η^2^ ≤ 0.01), medium (0.01 < η^2^ < 0.14), and large (η^2^ > 0.14) [15]. Sphericity (ε) was tested with Mauchly’s test (W). We applied the Greenhouse–Geisser (GG) correction in the case of sphericity assumption violation. If GG < 0.75, the *p*-value was corrected with the Greenhouse–Geisser method, otherwise, with the Huynh–Feldt method [16]. The *p*-values were adjusted using the False Discovery Rate (FDR) method for pairwise time analysis.

Statistics were computed using R-studio (Posit team 2022. RStudio, version 2025.09.2: Integrated Development Environment for R. Posit Software, PBC, Boston, MA, USA). All tests were performed with α = 0.05; a *p*-value < 0.05 was considered statistically significant.

Data are reported in tables and plots.

## 3. Results

### 3.1. Population and Procedure

Fifty-two patients underwent CEA with LA. Table 1 reported the main population and surgical characteristics. Briefly, 38 patients (73.1%) were male, with a median age of 74.0 years (q_1_–q_3_ 64.2–78.0 years). The three most common comorbidities were hypertension (51 patients, 98.1%), COPD (33 patients, 63.5%), and diabetes (31 patients, 59.6%).

LA was performed successfully in all patients, without pain at the skin incision or during surgery. Regarding sedation management, REMI was administered to 27 (51.9%) patients, while DEX was administered to 25 (48.1%). Nine patients (17.3%) required additional sedative administration for RASS ≥+2. The rates of bradycardia and hypotension were 19.2% (10 patients) and 13.5% (7 patients), respectively.

The median clamping time was 30.0 min (q_1_–q_3_ 25.0–35.0 min). A drop of >20% in rSO_2_ was observed in 13 patients (25.0%), and 10 patients (19.2%) had a positive awake test requiring shunt positioning. The shunt positioning led to a complete resolution of the neurological signs in all cases with normalization of the rSO_2_ value.

All surgical procedures were completed without conversion to general anesthesia. There were no perioperative strokes, myocardial infarctions, or deaths. All patients survived at hospital discharge without permanent neurological deficits.

### 3.2. REMI Group vs. DEX Group

Table 1 shows the results of the statistical comparison between the groups. The incidence of failed sedation was higher in the DEX than in the REMI group (32.0% vs. 3.7%, *p*-value = 0.020), as were the occurrence of bradycardia (36.0% in the DEX group vs. 3.7% in the REMI group, *p*-value = 0.009) and hypotension (28.0% in the DEX group vs. 0.0% in the REMI group, *p*-value = 0.011).

**Figure 1:**
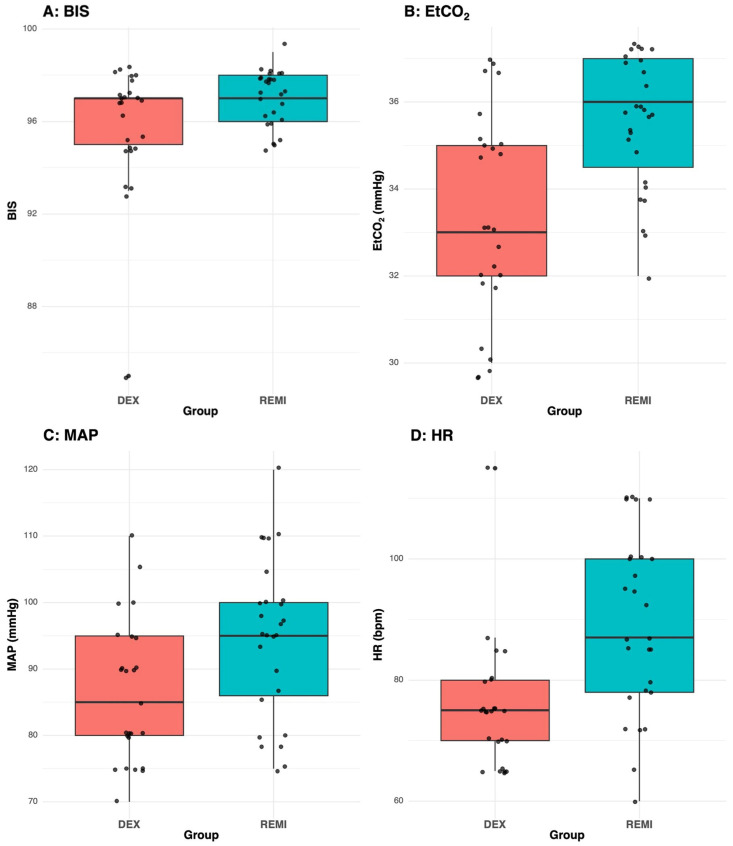
Box-plots. Panel (**A**): U-test = 230.5, *p*-value = 0.044. Panel (**B**): U-test = 169.0, *p*-value = 0.002. Panel (**C**): Levene test *p*-value = 0.872, *t* test = −3.755, df = 50, *p*-value < 0.001. Panel (**D**): U-test = 143.5, *p*-value < 0.001.

Baseline parameters (T_0_) showed significant differences between the groups. Despite the median BIS values (Figure 1A) being similar, the DEX group showed lower quartile values than the REMI group (q_1_–q_3:_ 95.0–97.0 and 96.0–98.0, respectively; *p*-value = 0.044). Furthermore, the DEX group showed lower EtCO_2_ (median 33.0 vs. 36.0 mmHg, *p*-value = 0.002, Figure 1B), MAP (mean 86.4 vs. 94.7 mmHg, *p*-value < 0.001, Figure 1C), and HR (median 75.0 vs. 87.0 bpm, *p*-value < 0.001, Figure 1D). No other differences were noted.

Univariate Firth’s penalized logistic regression showed that sedation with DEX was associated with increased risk of failed sedation (OR 8.58, CI_95%_ 1.70–85.81, *p*-value = 0.007), bradycardia (OR 10.17, CI_95%_ 2.05–101.21, *p*-value = 0.003), and hypotension (OR 22.30, CI_95%_ 2.46–2959.60, *p*-value = 0.003), but not with positive awake test (*p*-value = 0.589), and NIRS drop (*p*-value = 0.881). In the bivariable Firth’s penalized logistic regression models (Table 2), the direction of the associations observed in the univariable analyses was broadly maintained after adjustment for selected baseline (T_0_) covariates entered one at a time. Specifically, DEX remained associated with higher odds of sedation failure across models adjusted for BIS, EtCO_2_, MAP, or HR (*p*-values < 0.05). Similarly, DEX was associated with increased odds of intraoperative bradycardia (*p*-values < 0.05) and hypotension (*p*-values < 0.05). In contrast, no statistically significant associations were observed between regimen sedation and either a positive awake test or NIRS drop after adjustment (all *p*-values > 0.05).

### 3.3. Trend Analysis

Table 3 shows trends in BIS, HR, MAP, SpO_2_, and EtCO_2_.

**Figure 2:**
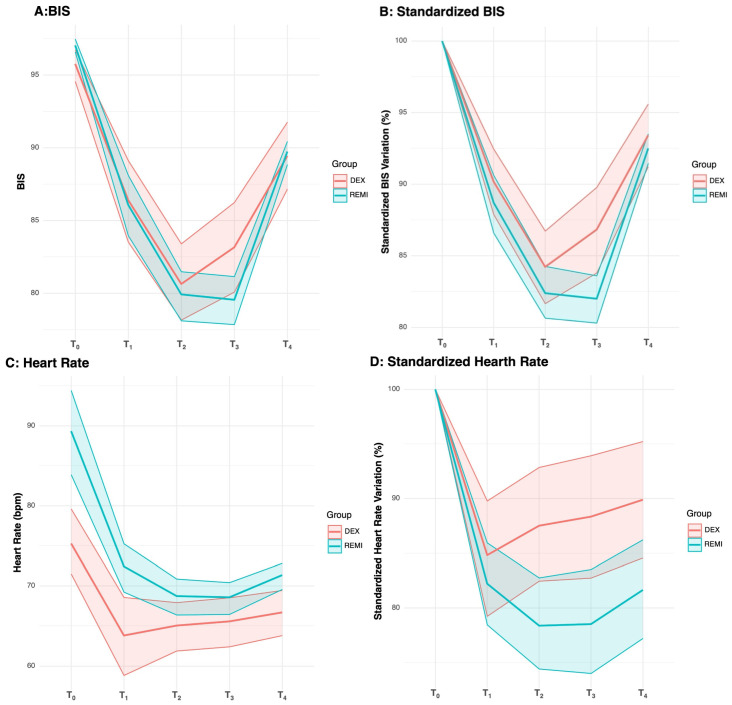
Trend lines for absolute and standardized for T_0_ value for BIS and HR. Panel (**A**): ε = 0.760, W-test *p*-value = 0.152. Difference between groups: *p*-value = 0.572, η^2^ = 0.003. Difference within groups: *p*-value < 0.001, η^2^ = 0.565, large effect. Group–time interaction: *p*-value = 0.027, η^2^ = 0.025, medium effect. Panel (**B**): ε = 0.760, W-test *p*-value = 0.152. Difference between groups: *p*-value = 0.065, η^2^ = 0.032. Difference within groups: *p*-value < 0.001, η^2^ = 0.604, large effect. Group–time interaction: *p*-value = 0.038, η^2^ = 0.027, medium effect. Panel (**C**): ε = 0.132, W-test *p*-value < 0.001. Difference between groups: *p*-value < 0.001, η^2^ = 0.128, medium effect. Difference within groups: GG *p*-value < 0.001, η^2^ = 0.304, large effect. Group–time interaction: GG *p*-value = 0.004, η^2^ = 0.051, medium effect. Panel (**D**): ε = 0.169, W-test *p*-value < 0.001. Difference between groups: *p*-value = 0.015, η^2^ = 0.067, medium effect. Difference within groups: GG *p*-value < 0.001, η^2^ = 0.256, large effect. Group–time interaction: GG *p*-value = 0.025, η^2^ = 0.030, medium effect.

BIS (Figure 2A,B) significantly varies over time, with different group patterns. However, the group’s main effect was not significant. Pairwise analysis showed that BIS at T_3_ differed between groups, both in absolute values (mean 79.6 in REMI group vs. 83.2 in DEX group, adjusted *p*-value = 0.014) and in variation (mean variation −18.0% in REMI group vs. −13.2% in DEX group, adjusted *p*-value < 0.001).

HR (Figure 2C,D) significantly varies between groups and over time. Moreover, the groups exhibit slightly different patterns, as evidenced by the significant group–time interaction. In pairwise analysis, the DEX group showed lower absolute HR values at T_0_ (mean 75.3 vs. 89.3 bpm, adjusted *p*-value < 0.001) and T_1_ (mean 63.8 vs. 72.4 bpm, adjusted *p*-value < 0.001) compared to the REMI group. For HR variation, pairwise analysis showed that the DEX group had lower percentage variation at T_2_ (mean variation −12.5% vs. −21.6%, adjusted *p*-value = 0.004), T_3_ (mean variation −11.6% vs. −21.5%, adjusted *p*-value = 0.002), and T_4_ (mean variation −10.1% vs. −18.3%, adjusted *p*-value = 0.009) compared to the REMI group.

**Figure 3:**
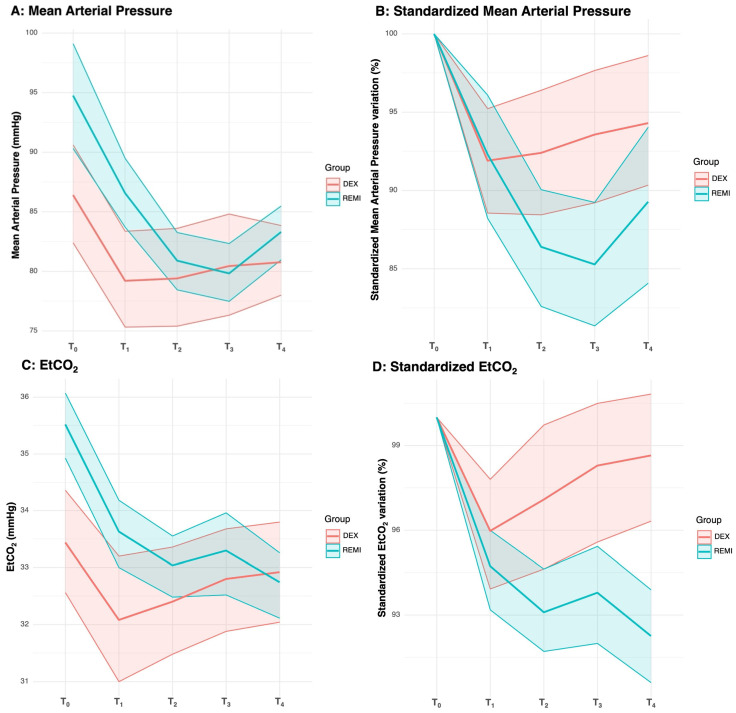
Trend lines for absolute and standardized for T_0_ value for MAP and EtCO_2_. Panel (**A**): ε = 0.402, W-test *p*-value < 0.001. Difference between groups: *p*-value = 0.070, η^2^ = 0.043. Difference within groups: GG *p*-value < 0.001, η^2^ = 0.154, large effect. Group–time interaction: GG *p*-value = 0.003, η^2^ = 0.035, medium effect. Panel (**B**): ε = 0.525, W-test *p*-value < 0.001. Difference between groups: *p*-value = 0.081, η^2^ = 0.039. Difference within groups: GG *p*-value < 0.001, η^2^ = 0.148, large effect. Group–time interaction: GG *p*-value = 0.004, η^2^ = 0.032, medium effect. Panel (**C**): ε = 0.780, W-test *p*-value = 0.213. Difference between groups: *p*-value = 0.059, η^2^ = 0.049. Difference within groups: *p*-value < 0.001, η^2^ = 0.095, medium effect. Group–time interaction: *p*-value < 0.001, η^2^ = 0.038, medium effect. Panel (**D**): ε = 0.779, W-test *p*-value = 0.210. Difference between groups: *p*-value < 0.001, η^2^ = 0.112, medium effect. Difference within groups: *p*-value < 0.001, η^2^ = 0.140, large effect. Group–time interaction: *p*-value < 0.001, η^2^ = 0.060, medium effect.

MAP (Figure 3A,B) significantly varies over time. The groups exhibit slightly different patterns, as evidenced by the significant group–time interaction, although the main effect of the group was not significant. In the pairwise analysis, the DEX group showed lower absolute MAP values at T_0_ (mean 86.4 vs. 94.7 mmHg; adjusted *p*-value = 0.001) and T_1_ (mean 79.2 vs. 86.6 mmHg; adjusted *p*-value = 0.005) compared with the REMI group. For MAP variation, pairwise analysis showed that the DEX group had lower MAP variation at T_2_ (mean variation −7.6% vs. −13.6%; adjusted *p*-value = 0.026) and T_3_ (mean variation −6.4% vs. −14.7%, adjusted *p*-value = 0.002) compared with the REMI group.

Two-way ANOVA for SpO_2_ (ε = 0.5154, W *p*-value < 0.001) showed no significant effect between groups (*p*-value = 0.148, η^2^ = 0.025), within time (GG *p*-value = 0.421, η^2^ = 0.008), and group–time interaction (GG *p*-value = 0.092, η^2^ = 0.017).

EtCO_2_ (Figure 3C,D) significantly varied over time, with different patterns, as evidenced by the significant group–time interaction. Differences between groups resulted significantly in the EtCO_2_ trend but not as absolute values (*p*-value < 0.001 and 0.059, respectively). In pairwise analysis, the DEX group showed lower EtCO_2_ values at T_0_ (mean 33.4 vs. 35.5 mmHg, adjusted *p*-value < 0.001) and T_1_ (mean 32.1 vs. 33.6 mmHg, adjusted *p*-value = 0.008) compared to the REMI group. For EtCO_2_ variation, the pairwise analysis showed that the DEX group had the lower values at T_2_ (mean variation −2.9% vs. −6.9%, adjusted *p*-value = 0.002), T_3_ (mean variation −1.7% vs. −6.2%, adjusted *p*-value < 0.001), and T_4_ (mean variation −1.3% vs. −7.7%, adjusted *p*-value < 0.001) compared to the REMI group.

## 4. Discussion

Our retrospective and explorative study evaluated the effectiveness and safety of DEX versus REMI for sedation during awake CEA under LA. In addition to its recognized advantages (sedation with minimal respiratory depression and preserved patient cooperation), DEX was associated with higher odds of sedation failure, bradycardia, and hypotension compared with REMI in both univariate and bivariate Firth-penalized logistic regression analyses. Significantly, regimen sedation was not associated with adverse neurological outcomes or an increased need for shunt placement. Our preliminary findings suggested that DEX, when used as a standalone sedative strategy, may be less suitable than REMI in this clinical setting.

The literature evidence regarding the effectiveness of DEX as a sole sedation agent in patients requiring vascular surgery is conflicting. In a randomized controlled trial (RCT), McCutcheon et al. [17] provided the strongest evidence for using DEX (initial loading dose 0.5 mcg/kg over 10 min followed by infusion at 0.2 mcg/kg/h) as additional sedation for CEA. Their data suggested beneficial pharmacological properties of DEX, including rapid patient arousal without respiratory depression, hemodynamic stabilizing effects, and mild analgesia. However, there was no mention of the failure rate and the requirement for different sedative administrations. In the awake test during CEA under LA, Lee et al. [18] showed that incorporating DEX (1 mcg/kg/h loading dose, followed by infusion at 0.4–0.6 mcg/kg/h) could reduce patient anxiety and enhance hemodynamic stability during surgery. In Bekker et al. [19], DEX (loading dose of 1 mcg/kg administered over 10 min, followed by a maintenance dose of 0.3 mcg/kg/h) provided satisfactory sedation and greater patient cooperation than in the control group. However, in the same study, the need for sedative administrations was 45% for fentanyl, 48% for midazolam, and 13% for propofol. These data suggested that DEX was ineffective as the sole sedation agent. In an RCT by Huncke et al. [20], DEX (1.0 or 0.5 mcg/kg load given over 10 min, followed by a maintenance infusion at 0.6 mcg/kg/h) was adequate as a sole sedative for approximately 50% of the patients. Additional midazolam was commonly administered to maintain target sedation, but the total dose was significantly lower during DEX infusions.

Our findings and the literature data underscore the necessity for anesthesiologists to have alternative sedation strategies readily available, suggesting that DEX may be more appropriate in combination with other sedatives rather than as monotherapy.

The trend analysis for BIS, HR, MAP, and EtCO_2_ revealed notable differences between and within groups. These effects were more pronounced when baseline standardized values were analyzed.

The BIS trend showed distinct patterns between the DEX and REMI groups. Notably, BIS appeared to decline more gradually under DEX, which may be consistent with its slower onset and progressive attainment of a steady sedative state compared with REMI. Importantly, these BIS findings should be interpreted as exploratory and hypothesis-generating only, and they are not intended to support causal inferences regarding sedation adequacy or “sedation failure.” Although BIS was not specifically designed to capture the pharmacodynamic effects of DEX, its use in this setting may provide a complementary, albeit imperfect, perspective on inter-individual variability in sedative responses. DEX is a highly selective α_2_-adrenergic receptor agonist that produces a qualitatively different sedative state than opioids or hypnotic agents, resembling natural sleep with preserved arousability and cooperation [21]. This distinctive profile may modulate BIS differently from traditional hypnotics (e.g., propofol), for which BIS was originally developed. The reliability of BIS during DEX sedation remains debated, and correlations with clinical sedation scales (e.g., MOAA/S) are only moderate [22]. Therefore, BIS may be considered an adjunct tool in awake CEA, but it should be interpreted cautiously and always integrated with clinical sedation assessments and the ongoing neurological evaluation.

HR and MAP demonstrated significant temporal changes in both groups. In the REMI group, HR and MAP showed larger baseline-normalized decreases over time compared with the DEX group, indicating a more pronounced average downtrend rather than a higher likelihood of crossing clinically relevant thresholds. These findings are consistent with prior reports suggesting that DEX is often associated with relatively stable hemodynamic profiles, whereas REMI may induce more rapid and noticeable fluctuations in HR and blood pressure due to its fast onset [23,24]. However, no statistically significant differences were observed in absolute HR and MAP values at ICA clamping (T_2_) and declamping (T_3_). This pattern may reflect the different pharmacokinetic profiles of the two agents: REMI has a very rapid onset (typically within 1–2 min) owing to high lipophilicity and fast tissue distribution [25], whereas DEX has a slower onset, with clinically appreciable sedation usually occurring 10–15 min after administration [26].

In our study, baseline HR and MAP were significantly lower in the DEX group, indicating clinically relevant baseline imbalance between treatment arms. Nevertheless, in our bivariable Firth-penalized models, DEX remained associated with bradycardia and hypotension even after separate adjustment for baseline (T_0_) HR and MAP, suggesting that the observed excess risk cannot be explained by baseline hemodynamics alone, although residual confounding cannot be excluded. The relationship between pre-infusion cardiovascular status and hemodynamic events during DEX infusion is multifactorial [27]. In particular, low baseline HR is a recognized risk factor for bradycardia after DEX initiation, and many studies exclude patients with baseline HR < 50–55 bpm because of the increased risk of severe bradycardia [28]. Likewise, baseline arterial pressure may influence the magnitude of MAP reduction; Wujtewicz et al. [29] reported that pre-treatment systolic blood pressure predicted a significant decrease in MAP following DEX administration. Importantly, a regimen may show larger mean relative reductions without generating more threshold events if baseline values are higher and/or if early dose adjustments prevent crossings; conversely, lower baseline HR/MAP or greater susceptibility may translate into more threshold events even with smaller mean relative changes. Taken together, these observations underscore the need to carefully consider baseline cardiovascular parameters, apply appropriate analytical adjustment, and ensure close hemodynamic monitoring during DEX infusion to anticipate and promptly manage potential adverse effects.

EtCO_2_ trends also diverged between the two groups, with significant differences within groups. EtCO_2_ monitoring can detect apnea earlier than SpO_2_, reducing the incidence of hypoxic events [30,31]. Despite REMI, as an opioid, being associated with a reduction in respiratory rate that can lead to respiratory depression [32], in the present study, the REMI group exhibited a more pronounced decrease in EtCO_2_. Notably, the SpO_2_ trends between the two groups did not differ significantly, suggesting that oxygenation remained stable and unaffected. This finding, combined with the EtCO_2_ trends, underscores that in a controlled and monitored environment, the risk of clinically significant hypoventilation in patients sedated with REMI appears to be more theoretical than actual. Beyond these observations, it is essential to consider the limitations of measuring EtCO_2_ in non-intubated patients. Abnormalities in the EtCO_2_ monitoring waveform can be observed, and EtCO_2_ measurements can be unreliable. Despite these limitations, our data suggest that REMI maintained adequate ventilation and oxygenation, reinforcing its safety profile as a sedative agent.

### Strengths and Limitations

This study has several strengths that enhance its relevance and applicability. It focuses on a specific and challenging clinical context, sedation during awake CEA, providing practical insights for balancing patient cooperation and procedural safety. By directly comparing DEX and REMI, the study offers a valuable head-to-head evaluation of two widely used sedatives, with findings grounded in real-world clinical practice. The comprehensive data collection, including hemodynamic, respiratory, and sedation depth parameters, allows for a thorough assessment of efficacy and safety. Using standardized protocols and trend analysis further strengthens the reliability and depth of the findings, highlighting temporal patterns in sedation effects. Patient safety is emphasized, with effective management of adverse events such as bradycardia and hypotension. Finally, by addressing a gap in the literature, this exploratory, hypothesis-generating study provides preliminary evidence and informs the design of future investigations to optimize sedation strategies for awake CEA.

This study has several limitations. First, its retrospective single-center design and small sample size preclude causal inference. Because treatment assignment was not randomized and allocation concealment was not feasible, residual confounding and selection bias cannot be excluded despite the alternating use of the two regimens. Second, baseline imbalances (HR, MAP, EtCO_2_, and BIS at T_0_) suggest potential confounding. These differences are consistent with allocation-related confounding (confounding by indication), as treatment assignment was not randomized and may have been influenced by patient- or session-level clinical considerations. Given the limited number of events, we intentionally avoided fully adjusted multivariable models to reduce overfitting; therefore, adjusted estimates should be considered exploratory and interpreted as associations rather than causal effects. Third, sedation failure was defined as clinically relevant agitation (RASS ≥ +2) requiring rescue sedation, a threshold chosen because it is incompatible with safe awake CEA. However, the definition of sedation failure was intrinsically time-sensitive and influenced by intraoperative workflow requirements (i.e., the need for rapid control of agitation to preserve surgical immobility and immediate neurological testing). Therefore, the slower onset and titration of DEX compared with REMI may have preferentially biased this endpoint, and in some cases, “failure” may have reflected the need for rapid intraoperative control rather than pharmacological inefficacy. Fourth, the absence of major complications reflects the context of a highly standardized institutional practice (single attending anesthesiologist and a single experienced vascular surgeon), which may limit generalizability; moreover, the study was underpowered for rare adverse events, and the lack of complications cannot be interpreted as evidence of equivalence between regimens. Finally, EtCO_2_ measurement in non-intubated patients is inherently susceptible to artifacts related to device fit, motion, and oxygen supplementation, which may have affected the accuracy of respiratory trends.

## 5. Conclusions

In this retrospective cohort of patients undergoing awake CEA, DEX-based sedation was associated with higher rates of threshold-defined sedation failure and hemodynamic adverse events (bradycardia and hypotension) compared with REMI. These findings should be interpreted cautiously, as baseline hemodynamic differences, patient selection, and nonrandom treatment allocation may have contributed to the observed between-group differences, and residual confounding cannot be excluded. Importantly, in this single-center experience, these events were generally manageable and were not associated with an apparent reduction in procedural success or primary perioperative outcomes.

Overall, our results are exploratory and hypothesis-generating and do not support definitive conclusions regarding the comparative suitability of DEX as a standalone sedative for awake CEA. Prospective studies with larger, better-balanced cohorts and robust strategies for confounding control (e.g., propensity-based methods and/or adequately powered multivariable models) are warranted to clarify comparative safety and effectiveness and to refine patient-tailored sedation protocols, particularly with respect to baseline cardiovascular profile and intraoperative titration requirements.

## Figures and Tables

**Table 1:** The main population characteristics, intraoperative and surgical management, and the difference between the REMI and DEX groups.

Variable		Overall (*n* = 52)		REMI Group (*n* = 27)	DEX Group (*n* = 25)	*p*-Value
		Result	Min–Max			
Sex, male (%)		38 (73.1%)	-	19 (70.4%)	19 (76.0)	0.885
Age (years)		74.0 [64.2–78.0]	45.0–86.0	71.0 [61.5–76.5]	78.0 [66.0–80.0]	0.106
Weight (kg)		75.5 ± 10.0	45.0–100.0	78.0 [74.0–80.0]	75.0 [70.0–80.0]	0.541
***Comorbidities***						
Hypertension (%)		51 (98.1%)	-	27 (100.0%)	24 (96.0%)	0.969
COPD (%)		33 (63.5%)	-	19 (70.4%)	14 (56.0%)	0.431
Diabetes (%)		31 (59.6%)	-	20 (74.1%)	11 (44.0%)	0.054
Coronary Disease (%)		17 (32.7%)	-	10 (37.0%)	7 (28.0%)	0.690
Previous TIA/Ictus (%)		13 (25.0%)	-	7 (25.9%)	6 (24.0%)	1.000
CABG/PTCA (%)		13 (25.0%)	-	8 (29.6%)	5 (20.0%)	0.631
Dilatative Cardiomyopathy (%)		12 (23.1%)	-	7 (25.9%)	5 (20.0%)	0.859
Chronic Renal Failure (%)		7 (13.5%)	-	5 (18.5%)	2 (8.0%)	0.482
ASA	II (%)	11 (21.1%)	-	5 (18.5%)	6 (24.0%)	0.886
	III (%)	41 (78.8%)	-	22 (81.4%)	19 (76.0%)	
Intraoperative management						
Failure sedation (%)		9 (17.3%)	-	1 (3.7%)	8 (32.0%)	0.020
Bradycardia (%)		10 (19.2%)	-	1 (3.7%)	9 (36.0%)	0.009
Hypotension (%)		7 (13.5%)	-	0 (0.0%)	7 (28.0%)	0.011
***Baseline Parameters (T*** **_0_** ***)***						
BIS		97.0 [95.0–98.0]	85.0–99.0	97.0 [96.0–98.0]	97.0 [95.0–97.0]	0.044
SpO_2_ (%)		100.0 [100.0–100.0]	97.0–100.0	100.0 [100.0–100.0]	100.0 [100.0–100.0]	0.626
EtCO_2_ (mmHg)		35.0 [33.0–36.2]	30.0–37.0	36.0 [34.5–37.0]	33.0 [32.0–35.0]	0.002
MAP (mmHg)		90.0 [80.0–100.0]	70.0–120.0	94.7 ± 12.0	86.4 ± 10.6	<0.001
HR (bpm)		79.0 [72.0–92.7]	60.0–115.0	87.0 [78.0–100.0]	75.0 [70.0–80.0]	<0.001
***Surgery***						
Clamping time (min)		30.0 [25.0–35.0]	20.0–40.0	30.0 [25.0–35.0]	30.0 [25.0–30.0]	0.138
Positive awake test (%)		10 (19.2%)	-	6 (22.2%)	4 (16.0%)	0.828
NIRS drop (%)		13 (25.0%)	-	7 (25.9%)	6 (24.0%)	1.000

Categorical data are expressed as absolute numbers and percentages (%). Continuous data were reported as median and first and third quartiles [q_1_–q_3_] or as mean ± standard deviation, depending on their distribution (tested by the Shapiro–Wilk test). Minimum (Min) and maximum (Max) values were also reported. The difference in categorical variables was tested with the χ^2^ test. The Student *t*-test (or Welch test for heteroscedasticity) or the Mann–Whitney test, according to data distribution, were used for continuous variables. All tests were performed with α = 0.05. A *p*-value < 0.05 was considered significant. Bold and italic formatting in this table are used for readability and visual hierarchy, to distinguish explanatory notes from the main table content.

**Table 2:** Firth’s penalized logistic regression (bivariable models) evaluating the association between DEX sedation and intraoperative outcomes, adjusted for baseline (T_0_) covariates.

Variable	Adjusted for BIS		Adjusted for EtCO_2_		Adjusted for Mean Arterial Pressure		Adjusted for Heart Rate	
	OR (CI_95%_)	*p*-Value	OR (CI_95%_)	*p*-Value	OR (CI_95%_)	*p*-Value	OR (CI_95%_)	*p*-Value
Sedation Failure	8.24 (1.56–84.01)	0.011	15.67 (2.64–175.76)	0.001	14.97 (2.39–189.57)	0.002	30.23 (3.16–1481.73)	0.001
Bradycardia	9.60 (1.86–97.40)	0.005	6.33 (1.06–67.8)	0.042	9.91 (1.86–104.18)	0.005	6.41 (1.14–68.79)	0.034
Hypotension	11.95 (1.13–1629.04)	0.038	18.25 (1.71–2505.57)	0.013	16.67 (1.76–2230.81)	0.010	31.71 (2.66–4678.26)	0.003
Positive awake test	0.73 (0.17–2.90)	0.659	0.97 (0.21–4.18)	0.968	0.97 (0.22–4.21)	0.965	0.95 (0.20–4.52)	0.943
NIRS drop	0.75 (0.20–2.74)	0.669	1.01 (0.25–3.94)	0.985	1.04 (0.28–3.89)	0.951	1.65 (0.39–8.10)	0.500

Each column reports the odds ratio (OR) with 95% confidence interval (CI_95%_) and *p*-value for DEX (vs. REMI) from separate bivariable models in which the sedative regimen and one baseline (T_0_) covariate (BIS, EtCO_2_, mean arterial pressure, or heart rate) were entered simultaneously.

**Table 3:** Trends analysis for absolute and standardized values.

Variable	Time	Absolute Values			T_0_ Standardized Values		
		REMI Group	DEX Group	Pairwise *p*-Value	REMI Group	DEX Group	Pairwise *p*-Value
**BIS**	**T_0_**	97.0 ± 1.2	95.8 ± 2.8	0.382	-	-	-
	**T_1_**	86.1 ± 5.3	86.4 ± 7.0	0.823	−11.3 ± 5.4	−9.8 ± 6.0	0.296
	**T_2_**	79.9 ± 4.7	80.6 ± 6.9	0.643	−17.6 ± 4.9	−15.8 ± 6.7	0.190
	**T_3_**	79.6 ± 4.5	83.2 ± 8.0	0.014	−18.0 ± 4.7	−13.2 ± 7.7	<0.001
	**T_4_**	89.7 ± 2.2	89.4 ± 5.9	0.837	−7.5 ± 2.7	−6.6 ± 5.7	0.513
**HR (bpm)**	**T_0_**	89.3 ± 14.7	75.3 ± 10.6	<0.001	-	-	-
	**T_1_**	72.4 ± 8.3	63.8 ± 12.8	<0.001	−17.8 ± 9.8	−15.2 ± 13.7	0.407
	**T_2_**	68.7 ± 5.9	65.0 ± 7.8	0.147	−21.6 ± 10.9	−12.5 ± 13.3	0.004
	**T_3_**	68.5 ± 5.4	65.6 ± 8.1	0.235	−21.5 ± 12.6	−11.6 ± 14.8	0.002
	**T_4_**	71.3 ± 4.4	66.7 ± 7.1	0.066	−18.3 ± 12.0	−10.1 ± 13.5	0.009
**MAP (mmHg)**	**T_0_**	94.7 ± 12.0	86.4 ± 10.6	0.001	-	-	-
	**T_1_**	86.6 ± 8.0	79.2 ± 10.5	0.005	−7.7 ± 10.5	−8.1 ± 8.1	0.876
	**T_2_**	80.9 ± 6.4	79.4 ± 10.6	0.561	−13.6 ± 10.3	−7.6 ± 10.4	0.026
	**T_3_**	79.8 ± 6.4	80.4 ± 11.2	0.807	−14.7 ± 10.5	−6.4 ± 11.0	0.002
	**T_4_**	83.3 ± 6.2	80.8 ± 8.0	0.323	−10.7 ± 12.7	−5.7 ± 11.0	0.061
**SpO_2_ (%)**	**T_0_**	99.6 ± 1.0	99.5 ± 1.1	0.500	-	-	-
	**T_1_**	99.6 ± 0.8	99.9 ± 0.6	0.195	0.0 ± 0.9	+0.4 ± 1.0	0.099
	**T_2_**	99.4 ± 1.0	100 ± 0.0	0.013	−0.2 ± 1.2	+0.5 ± 1.1	0.008
	**T_3_**	99.8 ± 0.5	99.8 ± 0.8	0.936	+0.1 ± 1.0	+0.3 ± 1.1	0.619
	**T_4_**	99.7 ± 0.7	99.8 ± 0.7	0.789	+0.1 ± 1.0	+0.3 ± 1.1	0.428
**EtCO_2_ (mmHg)**	**T_0_**	35.5 ± 1.5	33.4 ± 2.4	<0.001	-	-	-
	**T_1_**	33.6 ± 1.7	32.1 ± 2.7	0.008	−5.3 ± 4.0	−4.0 ± 5.2	0.335
	**T_2_**	33.0 ± 1.4	32.4 ± 2.4	0.267	−6.9 ± 4.1	−2.9 ± 6.5	0.002
	**T_3_**	33.3 ± 1.8	32.8 ± 2.4	0.387	−6.2 ± 4.5	−1.7 ± 6.2	<0.001
	**T_4_**	32.7 ± 1.5	32.9 ± 2.3	0.754	−7.7 ± 4.4	−1.3 ± 6.0	<0.001

Data are reported as mean ± standard deviation. The pairwise time difference was tested with the Student *t*-test, and the *p*-value was adjusted for the False Discovery Rate method. All tests were performed with α = 0.05. A *p*-value < 0.05 was considered significant. Bold formatting in this table is used to improve content readability.

## Data Availability

The data presented in this study are available on request from the corresponding author due to institutional and ethical restrictions, including patient privacy and data protection regulations.

## Abbreviations

The following abbreviations are used in this manuscript:

ANOVA	Analysis of Variance (two-way, repeated measures)
ASA	American Society of Anesthesiologists Physical Status
BIS	Bispectral Index
CABG	Coronary Artery Bypass Grafting
CEA	Carotid Endarterectomy
CI_95%_	95% Confidence Interval
CO	Cerebral Oximetry
COPD	Chronic Obstructive Pulmonary Disease
DEX	Dexmedetomidine
EtCO_2_	End-tidal Carbon Dioxide
FDR	False Discovery Rate (*p*-value adjustment)
GG	Greenhouse–Geisser (sphericity correction)
HF	Huynh–Feldt (sphericity correction)
HR	Heart Rate
ICA	Internal Carotid Artery
LA	Local Anesthesia
MAP	Mean Arterial Pressure
MOAA/S	Modified Observer’s Assessment of Alertness/Sedation
NIRS	Near-Infrared Spectroscopy
OR	Odds Ratio
PTCA	Percutaneous Transluminal Coronary Angioplasty
q_1_–q_3_	First and Third Quartile
RASS	Richmond Agitation–Sedation Scale
RCT	Randomized Controlled Trial
REMI	Remifentanil
rSO_2_	Regional Cerebral Oxygen Saturation
SD	Standard Deviation
SpO_2_	Peripheral Oxygen Saturation
STROBE	Strengthening the Reporting of Observational Studies in Epidemiology
W (Mauchly’s W)	Mauchly’s Test Statistic for Sphericity

## References

[1] Mendonca C.T., Fortunato J.A., Carvalho C.A., Weingartner J., Filho O.R., Rezende F.F., Bertinato L.P. (2014). Carotid endarterectomy in awake patients: Safety, tolerability and results. Rev. Bras. Cir. Cardiovasc..

[2] Yuksel V., Ozdemir A.C., Huseyin S., Guclu O., Turan F.N., Canbaz S. (2016). Impact of Surgeon Experience During Carotid Endarterectomy Operation and Effects on Perioperative Outcomes. Braz. J. Cardiovasc. Surg..

[3] Harky A., Chan J.S.K., Kot T.K.M., Sanli D., Rahimli R., Belamaric Z., Ng M., Kwan I.Y.Y., Bithas C., Makar R. (2020). General Anesthesia Versus Local Anesthesia in Carotid Endarterectomy: A Systematic Review and Meta-Analysis. J. Cardiothorac. Vasc. Anesth..

[4] Allain R., Marone L.K., Meltzer J., Jeyabalan G. (2005). Carotid endarterectomy. Int. Anesthesiol. Clin..

[5] Yastrebov K. (2004). Intraoperative management: Carotid endarterectomies. Anesth. Clin. N. Am..

[6] Glass P.S. (1996). Remifentanil: A Viewpoint by Peter SA Glass. Drugs.

[7] Ebner F.H., Trenti E., Baldinelli F., Natto M., Ebner H. (2008). Carotid endarterectomy: Comparing anesthesia in awakened and intubated patients with general anesthesia. Minerva Cardioangiol..

[8] Krenn H., Deusch E., Jellinek H., Oczenski W., Fitzgerald R.D. (2002). Remifentanil or propofol for sedation during carotid endarterectomy under cervical plexus block. Br. J. Anaesth..

[9] Bevilacqua S., Romagnoli S., Ciappi F., Lazzeri C., Gelsomino S., Pratesi C., Gensini G.F. (2010). Anesthesia for Carotid Endarterectomy: The Third Option. Patient Cooperation During General Anesthesia. Surv. Anesthesiol..

[10] Scimia P., Giordano C., Basso Ricci E., Petrucci E., Fusco P. (2018). The ultrasound-guided C2-C4 compartment block combined to dexmedetomidine sedation: An ideal approach for carotid endarterectomy in awake patients. Minerva Anestesiol..

[11] Gallego-Ligorit L., Vives M., Valles-Torres J., Sanjuan-Villarreal T.A., Pajares A., Iglesias M. (2018). Use of Dexmedetomidine in Cardiothoracic and Vascular Anesthesia. J. Cardiothorac. Vasc. Anesth..

[12] Reel B., Maani C.V. (2025). Dexmedetomidine. StatPearls.

[13] Kondov S., Beyersdorf F., Schollhorn J., Benk C., Rylski B., Czerny M., Harloff A., Siepe M. (2019). Outcome of Near-Infrared Spectroscopy-Guided Selective Shunting During Carotid Endarterectomy in General Anesthesia. Ann. Vasc. Surg..

[14] Terakado T., Marushima A., Koyama Y., Tsuruta W., Takigawa T., Ito Y., Hino T., Sato M., Hayakawa M., Ishikawa E. (2019). Effectiveness of Near-Infrared Spectroscopy (NIRO-200NX, Pulse Mode) for Risk Management in Carotid Artery Stenting. World Neurosurg..

[15] Cohen J. (2013). Statistical Power Analysis for the Behavioral Sciences.

[16] Lecoutre B. (1991). A correction for the $\tilde{\varepsilon }$ approximate test in repeated measures designs with two or more independent groups. J. Educ. Stat..

[17] McCutcheon C.A., Orme R.M., Scott D.A., Davies M.J., McGlade D.P. (2006). A comparison of dexmedetomidine versus conventional therapy for sedation and hemodynamic control during carotid endarterectomy performed under regional anesthesia. Anesth. Analg..

[18] Lee J., Huh U., Song S., Chung S.W., Sung S.M., Cho H.J. (2016). Regional Anesthesia with Dexmedetomidine Infusion: A Feasible Method for the Awake Test during Carotid Endarterectomy. Ann. Vasc. Dis..

[19] Bekker A.Y., Basile J., Gold M., Riles T., Adelman M., Cuff G., Mathew J.P., Goldberg J.D. (2004). Dexmedetomidine for awake carotid endarterectomy: Efficacy, hemodynamic profile, and side effects. J. Neurosurg. Anesthesiol..

[20] Huncke T.K., Adelman M., Jacobowitz G., Maldonado T., Bekker A. (2010). A prospective, randomized, placebo-controlled study evaluating the efficacy of dexmedetomidine for sedation during vascular procedures. Vasc. Endovasc. Surg..

[21] Huupponen E., Maksimow A., Lapinlampi P., Sarkela M., Saastamoinen A., Snapir A., Scheinin H., Scheinin M., Merilainen P., Himanen S.L. (2008). Electroencephalogram spindle activity during dexmedetomidine sedation and physiological sleep. Acta Anaesthesiol. Scand..

[22] Ki S., Lee D., Lee W., Cho K., Han Y., Lee J. (2022). Verification of the performance of the Bispectral Index as a hypnotic depth indicator during dexmedetomidine sedation. Anesth. Pain Med..

[23] Hu R., Liu J.X., Jiang H. (2013). Dexmedetomidine versus remifentanil sedation during awake fiberoptic nasotracheal intubation: A double-blinded randomized controlled trial. J. Anesth..

[24] Janatmakan F., Nassajian N., Jarirahmadi S., Tabatabaee K., Zafari M. (2021). Comparison of the Effect of Dexmedetomidine and Remifentanil on Pain Control After Spinal Surgery: A Double-Blind, Randomized Clinical Trial. Anesthesiol. Pain. Med..

[25] Cascone S., Lamberti G., Titomanlio G., Piazza O. (2013). Pharmacokinetics of Remifentanil: A three-compartmental modeling approach. Transl. Med. UniSa.

[26] Maze M., Angst M. (2004). Dexmedetomidine and Opioid Interactions: Defining the Role of Dexmedetomidine for Intensive Care Unit Sedation. Anesthesiology.

[27] Roberts D.J., Haroon B., Hall R.I. (2012). Sedation for critically ill or injured adults in the intensive care unit: A shifting paradigm. Drugs.

[28] Izumida T., Imamura T. (2022). Appropriate Strategy for Preventing Bradycardia-induced Cardiac Arrest by Dexmedetomidine. Intern. Med..

[29] Wujtewicz M., Twardowski P., Jasinski T., Michalska-Malecka K., Owczuk R. (2023). Evaluation of the Relationship between Baseline Autonomic Tone and Haemodynamic Effects of Dexmedetomidine. Pharmaceuticals.

[30] Hinkelbein J., Lamperti M., Akeson J., Santos J., Costa J., De Robertis E., Longrois D., Novak-Jankovic V., Petrini F., Struys M. (2018). European Society of Anaesthesiology and European Board of Anaesthesiology guidelines for procedural sedation and analgesia in adults. Eur. J. Anaesthesiol..

[31] Conway A., Tipton E., Liu W.H., Conway Z., Soalheira K., Sutherland J., Fingleton J. (2019). Accuracy and precision of transcutaneous carbon dioxide monitoring: A systematic review and meta-analysis. Thorax.

[32] Sun H.T., Xu M., Chen G.L., He J. (2018). Study of comparing dexmedetomidine and remifentanil for conscious sedation during radiofrequency ablation of hepatocellular carcinoma. Zhonghua Yi Xue Za Zhi.

